# Mean Activated Clotting Time of Patients Receiving Intravenous Heparin and Undergoing Primary Percutaneous Coronary Intervention for ST Elevation Myocardial Infarction

**DOI:** 10.7759/cureus.56867

**Published:** 2024-03-25

**Authors:** Abdul R Solangi, Ahmed Wahab, Abdul R Ansari, Muhammad Tahseen, Syed Haris M Zaidi, Jamil Muqtadir

**Affiliations:** 1 Cardiology, National Institute of Cardiovascular Diseases, Karachi, PAK; 2 Medicine, Ziauddin University, Karachi, PAK; 3 Nephrology, Liaquat National Hospital, Karachi, PAK; 4 Infectious Diseases, Ziauddin University, Karachi, PAK; 5 Infectious Diseases, Dr. Ziauddin Hospital, Karachi, PAK

**Keywords:** st-elevation myocardial infarction (stemi), anticoagulant therapy, percutaneous coronary intervention, heparin, activated clotting time

## Abstract

Introduction

The most prevalent cause of death is acute myocardial infarction (AMI). Primary percutaneous coronary intervention (PPCI) has replaced thrombolysis as the recommended therapeutic option for individuals with ST-segment elevation myocardial infarction (STEMI). However, more effective anticoagulation regimes are required for PCI due to the limitations of unfractionated heparin.

Objective

This study aimed to ascertain the connection between the mean activated clotting time and the risk of bleeding and infarcts in individuals receiving intravenous heparin during PPCI for STEMI.

Methods

This was a one-year prospective observational study carried out at the National Institute of Cardiovascular Diseases (NICVD), Karachi, Pakistan.

Results

The majority (70.15%) were male, with a mean age of 56.08 ± 8.92 years. Following PPCI, the average active clotting time (ACT) was 350.56 ± 39.62 seconds (range 255 to 453), compared to the pre-PPCI mean of 504.15 ± 38.98 seconds. ACT was considerably higher in female patients, smokers, and overweight patients. The mean ACT was not significantly higher in patients with hypertension (HTN) and dyslipidemia (DLD).

Conclusion

The ACT range in this investigation was 255 to 453 seconds, and there was no discernible relationship between ACT readings and problems related to bleeding and ischemia. To determine who is more at risk, bleeding risk models should be used and improved further before catheterization.

## Introduction

The leading cause of mortality in Western societies is acute myocardial infarction (AMI) [1]. Over half of Pakistani adult deaths are due to ischemic heart disease. Within 12 hours of symptom onset, patients with ST-segment elevation myocardial infarction (STEMI), persistent ST-segment elevation, or new or suspected LBBB should receive mechanical or pharmacological reperfusion [2]. Primary percutaneous coronary intervention (PPCI) has superseded thrombolysis as the recommended therapeutic option for patients with STEMI [3,4]. During percutaneous coronary intervention (PCI), the American College of Cardiology (ACC), American Heart Association (AHA), and European Society of Cardiology (ESC) advise administering intravenous unfractionated heparin [5,6]. Unfractionated heparin does have several drawbacks, including its potentially disruptive impact on coagulation, its limited therapeutic window, the possibility of platelet activation induction, the danger of thrombocytopenia, and the uncertainty surrounding the optimal levels of activated clotting time. Consequently, improved anticoagulation protocols are necessary for PCI [7,8]. In addition, the drug shows inadequate control over the release of von Willebrand factor, rebound thrombin generation after stopping, and prothrombotic features linked to platelet activation [9,10].

At present, a variety of anticoagulants are in use, including unfractionated heparin (UFH) [11]; low-molecular-weight heparins (LMWHs), such as enoxaparin [6]; and direct antithrombins, such as bivalirudin. In light of the present consensus, the dose of UFH in PCI should be guided by activated clotting time (ACT) [12]. Nonetheless, in spite of these suggestions, it is still unclear if peri-procedural ischemic and hemorrhagic consequences are associated with UFH effects as determined by ACT [13,14]. The weak association between UFH dose and ACT, the use of specific ACT targets specified by glycoprotein (GP) inhibitors IIb/IIIa, and the variety of ACT measurement equipment add to the complexity. Several earlier research and historical agreements recommend greater ACTs (at least 300 seconds) to prevent acute ischemia consequences, such as abrupt vascular closure, despite increased bleeding rates [15-17]. In a study comparing intravenous enoxaparin and intravenous UFH, an International Randomized Evaluation trial found that while ischemia events occurred when ACT values were less than 325 seconds, bleeding rose dramatically when those values were greater [8]. In one study, the mean ACT was 319.8 ± 1.3 seconds, with a range of 165 to 750 seconds. Fifty-two (51%) of the patients had ACT times less than 300 seconds, which is regarded as subpar. Only 12 patients (11.8%) had ACT levels between 300 and 350 seconds, which is considered optimum, whereas 38 patients (37.2%) had ACT levels over 350 seconds [18].

This passage discusses the importance of restoring blood flow during PCI for myocardial reperfusion in acute STEMI. It highlights that while PCI initially helps restore epicardial flow, it can lead to microvascular dysfunction. Primary angioplasty has become preferable to fibrinolysis due to its reduced risk of serious cardiovascular events [19,20]. However, there is limited data on ACT levels in PCI patients, which are crucial for balancing the risk of bleeding and thrombotic complications. The study aims to determine the average ACT levels to inform anticoagulant therapy decisions and establish a baseline for risk and benefit associated with unfractionated heparin (UFH) therapy.

## Materials and methods

An observational study with a prospective design was carried out by the National Institute of Cardiovascular Diseases (NICVD) in Karachi, Pakistan, over the course of a year. In order to establish the sample size, they utilized the average duration for the ACT, which was 319.8 ± 1.3 seconds, along with a confidence interval of 95% and a margin of error of 10%. The calculated sample size was 650. Lastly, the information was imported into the software used by the WHO [18].

Study population

All patients, regardless of gender, who were diagnosed with acute STEMI and presented with chest discomfort during the first 90 minutes after the diagnosis and were undergoing PPCI were included in this study. Patients undergoing fibrinolytic therapy, patients with chronic kidney disease, and patients with underlying heart failure were not included in the study. In addition, we did not include women who were pregnant in the research.

Data collection procedure

The NICVD's Ethical Review Committee gave its approval (ERC Ref. #136-77-62/11-12-2023) on December 11, 2023. All the experiments were done in line with the Declaration of Helsinki. Patients who satisfied the inclusion criteria were asked for their informed consent before they were allowed to participate in the trial. By taking blood samples, we were able to obtain a baseline ACT value. A Hemochron test was used to perform ACT on each patient after they had been transferred to the catheterization laboratory. This test was performed both before and after the surgery. For the purpose of carrying out the procedure, either the radial or femoral route was utilized. Within four to six hours of the completion of the treatment, the patients were taken to the critical care unit. Following that, the sheath of the artery was removed. After the injection of heparin, the mean ACT of patients who were undergoing PPCI for STEMI was determined. In order to control confounding variables and biases, it was helpful to strictly adhere to the inclusion and exclusion criteria. In order to record all of the information, a pre-made proforma was utilized.

Statistical analysis

We collected and analyzed all the data using IBM SPSS Statistics for Windows, Version 21.0 (released 2012, IBM Corp., Armonk, NY). Gender, diabetes mellitus (DM), hypertension (HTN), dyslipidemia (DLD), and smoking were examined using frequency and percentage analyses as qualitative variables. The mean +/-SD (standard deviation) was calculated for age, duration of chest discomfort, ACT before PPCI, and ACT after PPCI. In order to account for the effect modifier, parameters, such as age, gender, DM, HTN, diabetes-related lung disease, smoking, body mass index (BMI), and duration of chest discomfort, were individually stratified. To find out how these factors affected the result variable, we utilized the Student's t-test; a p-value of less than 0.05 was deemed significant.

## Results

The study comprised 650 patients receiving PPCI, with a mean age of 56.08 ± 8.92 years. Similarly, the mean weight, height, BMI, and duration of chest pain are shown in Table 1.

**Table 1:** Descriptive statistics of characteristics of the patients (n = 650) CI: confidence interval, n: number of patients

Variables	Mean	Std. deviation	95% CI for mean		Median	Interquartile range
			Lower bound	Upper bound		
Age (years)	56.08	8.92	55.39	56.77	58	17
Height (cm)	163.14	10.35	162.34	163.93	165	16
Weight (kg)	71.19	9.37	70.47	71.91	70	16
BMI (kg/m^2^)	26.93	4.11	26.62	27.25	26.37	6.63
Duration chest pain	61.23	13.07	60.22	62.24	60.00	15

Of them, 70.15% were men, and almost 58% were overweight or obese. There were cases of 29.54% DLD, 63.08% HTN, and 67.54% diabetes; 34.15% of the 650 patients smoked, who were all men.

Patients receiving intravenous heparin during PPCI for STEMI and their mean ACT are shown in Table 2. Before PPCI, the mean baseline ACT was 504.15 ± 38.98 seconds (range 424 to 570), while after PPCI, the mean ACT was 350.56 ± 39.62 seconds (range 255 to 453).

**Table 2:** Mean ACT of patients undergoing PPCI for STEMI who received intravenous heparin CI: confidence interval

		Activated Clotting Time (Sec) (ACT)	
		Before PPCI (baseline)	After PPCI
Mean		504.15	350.56
Std. deviation		38.98	39.62
95% CI for the mean	Lower bound	501.15	347.50
	Upper bound	507.15	353.61
Minimum		424	255
Maximum		570	453

Table 3 compares the means of ACT values in various subgroups using stratification analysis; the mean ACT was not significant for other age groups. Table 4 and Table 5 demonstrate that the mean ACT was considerably higher in female patients, smokers, and overweight/obese patients. By contrast, Table 5 indicates that the mean ACT was not significantly higher in patients with HTN and DLD. Table 6 suggests that the duration of chest pain (within the investigated range of less than or more than 50 minutes) does not significantly affect the ACT before PPCI.

**Table 3:** Comparison of the mean ACT of patients undergoing PPCI among different age groups PPCI: primary percutaneous coronary intervention

PPCI	Age group (years)	n	Activated clotting time (sec)		P-value
			Mean	Std. deviation	
Before	<=40	19	497.47	45.09	0.72
	41 to 50	170	506.30	40.51	
	51 to 60	258	502.95	38.96	
	>60	203	504.50	37.25	
After	<=40	19	349.26	37.62	0.38
	41 to 50	170	354.99	43.17	
	51 to 60	258	349.59	38.21	
	>60	203	348.19	38.44	

**Table 4:** Comparison of the mean ACT of patients undergoing PPCI between males and females PPCI: primary percutaneous coronary intervention

PPCI	Gender	n	Activated clotting time (sec)		P-value
			Mean	Std. deviation	
Before	Male	456	502.24	36.88	0.06
	Female	194	508.65	43.29	
After	Male	456	348.01	37.06	0.012
	Female	194	356.54	44.61	

**Table 5:** Comparison of the mean ACT of patients undergoing PPCI between the presence and absence of risk factors n: number of patients, PPCI: primary percutaneous coronary intervention, BMI: body mass index

PPCI	Risk factors		Activated clotting time (sec)		P-value
		n	Mean	Std. deviation	
	Diabetes mellitus				
Before	Yes	439	504.56	38.63	0.69
	No	211	503.30	39.78	
After	Yes	439	346.35	38.20	0.0005
	No	211	359.30	41.15	
	Hypertension				
Before	Yes	410	502.59	40.19	0.18
	No	240	506.83	36.75	
	Yes	650	504.15	38.98	0.91
	No	410	350.43	41.35	
	Dyslipidemia				
Before	Yes	192	504.64	43.07	0.84
	No	458	503.95	37.18	
After	Yes	650	504.15	38.98	0.74
	No	192	349.75	39.30	
	Smoking				
Before	Smoker	222	501.96	38.59	0.30
	Non-smoker	428	505.29	39.18	
After	Smoker	650	504.15	38.98	0.0005
	Non-smoker	222	342.24	35.55	
	BMI				
Before	Normal	273	500.53	38.55	0.056
	Overweight	200	516.37	39.60	
	Obese	177	495.94	35.72	
After	Normal	273	347.60	33.87	0.012
	Overweight	200	355.14	37.22	
	Obese	177	349.93	49.11	

**Table 6:** Comparison of the mean ACT of patients undergoing PPCI between the duration of chest pain PPCI: primary percutaneous coronary intervention

PPCI	Duration of chest pain	n	Activated clotting time (sec)		P-value
			Mean	Std. deviation	
Before	≤50 min	173	500.26	37.46	0.12
	>50 min	477	505.56	39.46	
After	≤50 min	650	504.15	38.98	0.61
	>50 min	173	351.87	38.00	

## Discussion

The mainstay of care for coronary artery disease patients is PCIs. However, because of their invasive nature and the need for anticoagulants, bleeding problems represent a significant peri-procedural risk. The most popular invasive therapeutic cardiac technique for treating ischemic heart disease is PCI. Since Gruntzig published the first account of coronary angioplasty in humans, the procedure, tools, and related drugs have undergone remarkable development, which has resulted in notable reductions in peri-procedural problems [21,22]. Intravenous UFH has been the principal antithrombotic medication for the prevention of peri-procedural ischemic problems ever since PCI was developed [23]. A sufficient dosage of UFH efficiently inhibits the production of thrombin linked to balloon-induced vascular damage [24]. The ACT is a quick "point of care" test for dose individualization, and UFH is still a popular choice despite the ongoing development of antithrombotic therapies because it is inexpensive and readily available and has an antagonist that can quickly reverse antithrombin activity. Therefore, the majority of patients undergoing PCI should be able to achieve an appropriate dose of anticoagulation given the abundance of expertise connected with the therapeutic use of this drug. Sufficient dosage of UFH efficiently inhibits the production of thrombin linked to balloon-induced vascular damage

We included in our analysis 560 patients undergoing PPCI; their average age was 56.08 ± 8.92 years. Seventy-one percent of them were men. The AHA reports that women make up 34% of patients receiving PCI and 42% of patients hospitalized for AMI [25].

The recommended antithrombin during PCI has historically been UFH; an ACT of 250-300 is ideal [26]. It has been shown that an ACT of more than 350 seconds increases the risk of ischemia sequelae, including MI, mortality, and revascularization, in addition to the danger of bleeding [27]. The mean ACT of patients receiving intravenous heparin and undergoing PPCI for STEMI was 504.15 ± 38.98 seconds (range 424 to 570) prior to PPCI and 350.56 ± 39.62 seconds (range 255 to 453) following PPCI in our survey. Our results align with earlier research that found that smoking, female gender, and higher BMI independently predicted a higher mean of ACT in patients receiving intravenous heparin after PPCI for STEMI [28,29]. Our study found that the mean ACT was not significantly different in hypertensive persons, people with hyperlipidemia, or age groups. On the other hand, worldwide data indicate that individuals with elevated mean ACT are more prone to possess a past record of DM, HTN, or hyperlipidemia [30].

Despite this, there are some limitations to the study. One center (NICVD) in Karachi was the location of the research. Such variations in practices, demographics, or hospitals restrict the applicability of the results to external populations or hospitals. The interpretation of the data may also be impacted by the absence of blinding for those conducting the analysis. They might unwittingly favor particular outcomes when analyzing ACT measurements if they had knowledge of pre-existing conditions or post-procedural outcomes.

## Conclusions

Our study highlights the complexity of managing anticoagulation during PPCI for STEMI. Although we found no significant correlation between mean ACT and bleeding or ischemic events, our findings emphasize the importance of personalized anticoagulation strategies. Moving forward, clinicians should prioritize individualized approaches to anticoagulation management, considering patient-specific factors and procedural nuances. Collaboration and ongoing research are essential for refining anticoagulation protocols and improving outcomes in patients undergoing PPCI for STEMI.
